# Immunochemical Fecal Occult Blood Test for Detection of Advanced Colonic Adenomas and Colorectal Cancer: Comparison with Colonoscopy Results

**DOI:** 10.1155/2013/384561

**Published:** 2013-11-11

**Authors:** Bianca Rosa Viana Freitas, Cristiane Kibune Nagasako, Celia Regina Pavan, Sônia Letícia Silva Lorena, Fabio Guerrazzi, Cláudio Saddy Rodrigues Coy, Maria de Lourdes S. Ayrizono, Maria Aparecida Mesquita

**Affiliations:** ^1^Disciplina de Gastroenterologia, Departamento de Clínica Médica, Universidade Estadual de Campinas, Rua Tessália Vieira de Camargo 126, Campinas 13083-887, Brazil; ^2^Gastrocentro, Universidade Estadual de Campinas, Rua Carlos Chagas 420, Campinas 13083-878, Brazil; ^3^Departamento de Cirurgia, Universidade Estadual de Campinas, Rua Tessália Vieira de Camargo 126, Campinas 13083-887, Brazil

## Abstract

*Background*. Fecal immunochemical tests (FITs) have been used for colorectal cancer (CRC) screening in several countries. There is lack of information concerning diagnostic performances of this method in Brazil. *Methods*. Patients scheduled for elective colonoscopy provided one stool sample one week before colonoscopy. The accuracy of a qualitative FIT for detection of CRC and advanced adenomas was determined. *Results*. Overall 302 patients completed the study. Among them, 53.5% were high risk patients referred for screening or surveillance. Nine (3%) CRCs and 11 (3.6%) advanced adenomas were detected by colonoscopy. Sensitivity and specificity for CRC were, respectively, 88.9% and 87.6%. For advanced adenomas, sensitivity was 63.6% and specificity 87.6%. *Conclusion*. Our results showed good sensitivity and specificity of the FIT for detecting advanced neoplasias. This method may be a valuable tool for future screening programs in Brazil.

## 1. Introduction

Colorectal cancer (CRC) is a major cause of cancer morbidity and mortality worldwide [1, 2]. Over the last years there has been an increase in the reported incidence in Brazil, with estimates for 2012 of 26.2 new cases/100.000 for men and 25.6/100.000 for women in the regions with higher incidence rates [3]. In addition, analysis of CRC mortality in five Brazilian capitals showed an increasing and constant trend over the period of 1980–2009 [4]. 

The adenoma-carcinoma pathway is considered to be responsible for the majority of CRC [5]. Recent data from a long-term follow-up study showed that removal of adenomatous polyps was associated with a 53% reduction in mortality from CRC [6]. Considering the decrease in CRC incidence and mortality associated with population screening for CRC and precancerous lesions [7] guidelines in several countries recommend that adults from the general population at average risk should start screening at 50 years of age [1, 8, 9]. One of the proposed screening strategies is the survey with high sensitivity (≥70%) fecal occult blood test (FOBT). 

The traditional method to detect fecal occult blood has been the guaiac-based test (G-FOBT) that detects the peroxidase-like activity of hemoglobin. Although G-FOBT screening has been shown to reduce CRC incidence and mortality [7], the sensitivity of this method in detecting CRC and advanced adenomas has been shown to be low [10]. Another disadvantage of the G-FOBT is that it requires dietary restriction, since it is not specific for human blood and can produce false-positive results when red meat and fruits or vegetables containing peroxidase are ingested. In addition, bleeding in the upper gastrointestinal tract secondary to aspirin and nonsteroidal anti-inflammatory drugs can also produce false-positive tests. 

More recently, the replacement of G-FOBT by immunochemical fecal occult blood tests (FITs) has gained acceptance in the literature. Many authors now recommend that FIT should substitute G-FOBT for CRC screening [11]. FITs use antibodies directed against human hemoglobin, are highly specific for detecting human blood of colonic origin, and thus are not affected by diet or medications. In addition, several studies have demonstrated the superior diagnostic performance of FITs in detecting both colonic adenomas and cancers in comparison with standard G-FOBT [12–14]. 

Considering the lack of information regarding the diagnostic accuracy of FIT for advanced neoplasias in Brazil, the aim of the present study was to assess prospectively the sensitivity and specificity of a FIT in detecting CRC and advanced adenomas using colonoscopy as the reference test.

## 2. Subjects and Methods

All consecutive patients scheduled for elective colonoscopy at our university hospital endoscopy unit from July 2009 to July 2010 were invited to bring a stool sample on the day of the educational session about colonoscopy, which takes place one week before the exam. All patients who underwent colonoscopy and provided the stool sample were initially included in the study. Those with incomplete colonoscopy (which did not reach the cecum) and noncancerous bleeding lesions at the time of colonoscopy, such as inflammatory bowel disease or bleeding hemorrhoids, were excluded from the analysis. 

The study was approved by the Ethics Committee of our institution.

### 2.1. Colonoscopy and Histopathological Analysis

Before colonoscopy a questionnaire containing clinical and epidemiological data of the patient was completed. Colonoscopies were performed or supervised by experienced endoscopists who were blinded to the FIT results. 

Polyps identified at the time of the exam were characterized by number, size, colonic location (proximal or distal to the splenic flexure), and endoscopic appearance. All polyps were removed and sent for histological analysis. Adenomas were classified by number, size, location, and histologic characteristics (tubular, tubulovillous, or villous). 

Advanced adenoma was defined as either an adenoma sized >1 cm, or with villous or tubulovillous pattern, or with severe dysplasia [15]. 

For the purpose of analysis, in those cases with multiple polyps classification was based on the most histologically advanced lesion. Patients with diverticular diseases or nonbleeding hemorrhoids were considered as having normal colonoscopic findings. 

### 2.2. Fecal Immunochemical Test

The FIT used in the current study was the Feca-Cult One Step Test (Alamar Tecno Científica Ltda) which is an immunochromatographic test for the qualitative determination of human hemoglobin in feces. The manufacturer's quoted cutoff hemoglobin concentration is 0.2 **μ**g Hb/mL. FIT was performed in a single sample taken from a bowel movement one week before colonoscopy. All tests were performed by the same investigator, and borderline positive results (faint bands) were interpreted as positive. 

### 2.3. Statistical Analysis

CRCs and advanced adenomas were analysed separately. Continuous variables were reported as mean ± SD and categorical variables were reported as percentages. Comparisons of the results were performed by the Mann-Whitney *U* test, Student's *t*-test, and Fisher's exact test as appropriate. The diagnostic value of FIT for detecting advanced adenomas and CRCs was assessed by calculating the sensitivity and the specificity of the test. All statistical analyses were carried out using SPSS version 20.0 for Windows (SPSS, Inc., Chicago, IL, USA). *P* values < 0.05 were considered to be statistically significant. 

## 3. Results

**Figure 1:**
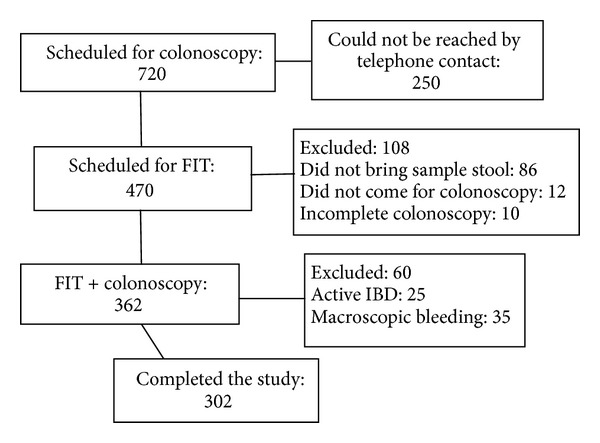
Flow chart of entry into the study.

Overall 302 patients were included in the study (Figure 1). Demographic characteristics of the study population are summarized in Table 1. Mean age was 56 ± 14 years, 73.5% were ≥50 years, and 64.2% were females.

Family history of CRC affecting one or two first-degree relatives, diagnosed at age ≥50 years in all cases, was reported by 36 (11.9%) patients.

The main indications for colonoscopy are shown in Table 2. It can be seen that 42% of patients were referred for colonoscopy because of symptoms, while 15.5% were high risk patients referred for screening and 38% for surveillance (polyps or previous surgery for CRC).

### 3.1. Colonoscopy Findings

Table 3 shows the colonoscopy findings in the study population. CRC was found in 9 patients (3%) whereas advanced adenomas were detected in 11 patients (3.6%). The mean age of patients with CRC was 68 ± 10 years (53–78 years) and 89% were females. All cases were adenocarcinomas, and 55.6% were well-differentiated tumors. In 6 patients (66.7%) the tumor was located in the distal colon. Two patients (22.2%) had a family history of CRC. Eight of them had no previous colonoscopy, while one had one adenoma in a previous examination. 

The mean age of patients with advanced adenomas was 65 ± 13 years, and 63.6% were females. The location of the lesions was the distal colon in 63.6% of the cases. Advanced adenomas size ranged from 12 to 40 mm, and the mean number of lesions was 3 ± 2. In 6 patients (55%), the adenomas were of the villous type. Two patients underwent colonoscopy because of polyps in previous exams. None of them had family history of CRC.

### 3.2. FIT Results

**Figure 2:**
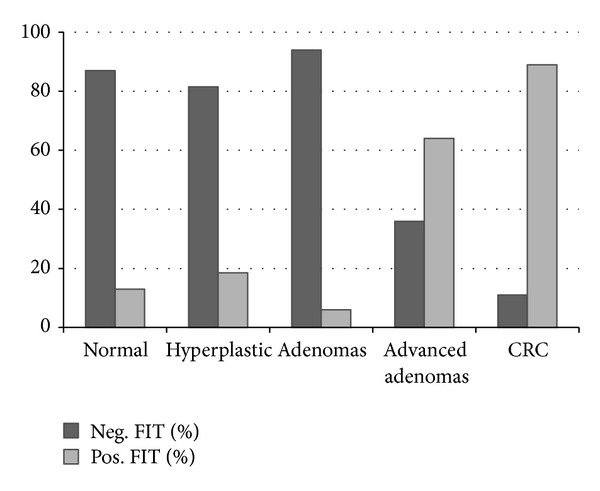
FIT positivity in relation to the colonoscopic findings.

In 50 patients (16.5%) the FIT was positive (borderline positive in two cases).  Figure 2 illustrates the positivity rate of the test in relation to the colonoscopic findings: 16.7% in patients with normal colonoscopy, 18.5% in hyperplastic polyps, 5.5% in adenomas, 63.6% in advanced adenomas, and 88.9% in CRC. There was a statistically significant association (*P* = 0.001) between FIT positivity and advanced adenomas and CRC.

### 3.3. FIT Performance

Table 4 shows the FIT performance in detecting advanced adenomas and CRC. FIT sensitivity for CRC was 88.9%, whereas specificity was 87.6%. For advanced adenomas, the test sensitivity was 63.6% and the specificity 87.6%.

The predictive positive value (PPV) for CRC and advanced adenoma was 18.6% and 16.7%, respectively, while the correspondent negative predictive value (NPV) was 99.6% and 98.4%.

## 4. Discussion

In this colonoscopy-controlled study we assessed the sensitivity and specificity of a qualitative FIT for detection of CRC and advanced adenomas. The sensitivity of 88.9% and specificity of 87.6% for CRC are within the range of 70–100% sensitivity and 80–97% specificity reported in other studies using FITs [16–18]. For example, three studies that used the quantitative test OC-Sensor reported the sensitivity and specificity for CRC as 80%/89% [19], 84.6%/89.8% [12], and 100%/91.7% [18]. 

The qualitative test is a chromatographic method, and the results are based on visual interpretation. Qualitative FIT permits simple, on-site analysis, does not require specific laboratory equipment, and therefore is less expensive than the automated quantitative FIT. However, the use of qualitative FITs has some disadvantages in relation to the quantitative method. One of them is the possibility of interobserver variation in the analysis of the results, particularly in those cases of faint bands. The comparison of different qualitative tests showed variations in the frequency of faint bands, which may contribute to differences in the positivity results among tests [20]. According to manufacturers, faint bands should be regarded as borderline positive results. However, the analysis of several qualitative tests showed that, for some of them, borderline results should be rated negative rather than positive [21]. 

In addition, previous studies have shown great differences in the positivity rates of different qualitative FITs, which were mainly related to different thresholds for detection of hemoglobin in stool [21, 22]. At the same time, there was a great variation in sensitivity and specificity for advanced neoplasias, which was strongly related to the positivity rate of the tests, indicating that lower cutoff values of fecal hemoglobin required to generate a positive result would render a higher test sensitivity. Therefore, test characteristics such as low frequency of faint bands and low cutoff value of fecal hemoglobin should be taken into account when selecting a qualitative test, reinforcing the importance of validation studies before implementation of the test in population-based screening. 

Our study is also in agreement with those showing the superior performance of FIT in detecting CRC in comparison with advanced adenomas [23]. It has been proposed that multiple rounds, such as annual FIT testing, are likely to detect many of the lesions missed on initial screens before they progress to CRC [24]. Computed simulations indicated that five rounds of testing would increase the sensitivity for advanced adenomas to the acceptable value of 81% [19].

Our results are based on the analysis of one fecal sample. It has been suggested that FIT sensitivity increases with the number of samples tested, such as 2- or 3-day analysis of fecal samples, specially for advanced adenomas [25]. However, other authors have shown that double sampling of FIT was not superior to single sampling for detection of advanced adenomas and CRC [26]. Further studies are necessary to clarify this issue.

The design of the present study allowed the direct calculation of sensitivity and specificity of the FIT, since all patients underwent colonoscopy. This is an advantage in relation to the studies of screening populations, in which only subjects who test positive are referred for colonoscopy. Sensitivity and specificity are not influenced by the prevalence of disease, as confirmed in a previous study showing similar FIT results in screening and referral patients [27]. 

On the other hand, predictive values are determined by sensitivity, specificity, and also the prevalence of the disease. The prevalence of advanced neoplasias in our study group composed of patients at average risk along with those at high risk for CRC may be higher than that seen in average risk screening population, and consequently, the test predictive positive value may be lower in the screening population. That means that a larger proportion of those with positive screening tests will be found not to have the disease upon colonoscopic examination.

A pilot study was held in a small city located in the state of São Paulo from 2006 to 2007, using a qualitative FIT (Hemosure) for the screening of residents aged over 40 years and at average risk for CRC [28]. Those with positive tests were invited to undergo colonoscopic examination. Overall, 3,640 tests, 43.7% of the target population, were analyzed. Results were positive in 390 (10.7%) exams. Colonoscopy was performed in 212 patients with positive FIT, showing polyps in 59 patients and adenocarcinoma in nine. All kits and colonoscopes were donations, and therefore cost analysis could not be performed. 

 Nowadays there is no public health policy for a nationwide CRC screening in Brazil. The implementation of a screening program in our country is highly dependent on public resources and therefore is critically affected by economic aspects. For example, the health department of the city of São Paulo, the most populated city in Brazil, recently concluded that at the moment it is not possible to implement a population screening for CRC in the city [29]. Considering the estimated target population (age 50–75 years) for 2011 as about two million people and a 50% participation rate, the use of biennial FIT (one sample) would require 550,000 kits and 55,000 colonoscopic examinations per year (considering the positivity rate of the test as 10%), which is more than twice the number of examinations performed at present. No cost-benefit analysis was performed. The evaluation has also highlighted the need for pilot studies to assess feasibility and cost effectiveness of regional screening programs. Other aspects that should be addressed in future studies include the choice between qualitative or quantitative FITs and annual or biennial screening.

In conclusion, the FIT showed good sensitivity and specificity for the detection of advanced neoplasia in our study population, indicating that this method may be a valuable tool for future screening programs in Brazil. 

## Figures and Tables

**Table 1:** Demographic characteristics of the study population.

Characteristics	*N* (%)
Age in years (mean ± SD)	56 ± 14
<50	80 (26.5)
≥50	222 (73.5)
Gender	
Females	194 (64.2)
Males	108 (35.8)
Personal history of CRC	80 (26.5)
Family history of CRC	36 (11.9)

**Table 2:** Main indications for colonoscopy in the study population.

Indications	*N* (%)
Symptoms	126 (42)
(i) Altered bowel habits	49 (38.8)
Diarrhea	27 (21.4)
Constipation	19 (15)
Alternating bowel habit	3 (2.4)
(ii) Abdominal pain	28 (22.2)
(iii) Anemia	16 (12.7)
(iv) Weight loss	9 (7.2)
(v) More than one of the symptoms above	16 (12.7)
(vi) Other symptoms	12 (6.4)
Screening (high risk patients)	47 (15.5)
(i) Inflammatory bowel disease	19 (40.4)
(ii) Family history of CRC	14 (29.8)
(iii) Previous G-FOBT +	6 (12.8)
(iv) Others	8 (17)
Surveillance	115 (38)
(i) Personal history of CRC	80 (69.6)
(ii) Polyps	35 (30.4)
Others	14 (4.5)
Total	**302 (100)**

**Table 3:** Colonoscopy findings in the study population.

Colonoscopy findings	*N* (%)
Normal	157 (52)
Polyps	73 (24)
Hyperplastic	27 (9)
Adenoma	35 (11.4)
Advanced adenoma	11 (3.6)
Diverticular disease	44 (14.6)
Inflamatory bowel disease	17 (5.7)
CRC	9 (3)
Others	2 (0.7)
Total	**302 (** **100** **)**

**Table 4:** FIT performance in detecting CRC and advanced adenomas.

	CRC (*n* = 9)	Advanced adenomas (*n* = 11)
Sensitivity % (IC 95%)	88.9 (52–99)	63.6 (31–89)
Specificity % (IC 95%)	87.6 (83–91)	87.6 (83–91)
Positive predictive value % (IC 95%)	18.6 (8–33)	16.7 (7–31)
Negative predictive value % (IC 95%)	99.6 (97–99)	98.4 (96–99)
